# From dysbiosis to precision medicine: targeting the microbial–metabolic axis in IBD management

**DOI:** 10.3389/fcimb.2026.1826972

**Published:** 2026-05-22

**Authors:** John K. Giju, Subin John, Amrutha Sivadas, Meera Prabhakar, Krishnakripa K., Damu Sunilkumar, Bipin G. Nair, Sanjay Pal, Vidhya Prakash

**Affiliations:** 1 School of Biotechnology, Amrita Vishwa Vidyapeetham, Kollam, Kerala, India; 2 The Ohio State University Wexner Medical Center and Comprehensive Cancer Center, Columbus, OH, United States

**Keywords:** Crohn’s disease, gut dysbiosis, intestinal homeostasis, machine learning, metagenomics, probiotics, SCFA (short-chain fatty acid), ulcerative colitis

## Abstract

Inflammatory bowel disease (IBD) is a chronic relapsing inflammatory condition that has a rapidly changing global epidemiology. IBD has been traditionally viewed as a primary immune system dysfunction, but emerging evidence more accurately describes IBD as a perturbance of the intricate balance between host immunity, the intestinal microbiome, and intestinal metabolism. Although genetic and environmental components have long been recognized as contributors, accumulating evidence increasingly highlights the pivotal role of microbial dysbiosis in the pathogenesis of IBD. In patients with IBD, intestinal dysbiosis, which is often characterized by reduced Firmicutes and increased pro-inflammatory bacteria, triggers a cascade of pathogenic events. These pathogenic events include impaired epithelial barrier function, dysregulated immune activation against luminal antigens, and immune reprogramming. Central to these processes are functional changes in microbial metabolism, particularly in pathways involving short-chain fatty acids (SCFAs), bile acids, and redox homeostasis, which critically contribute to the development of chronic mucosal inflammation. The current therapeutic backbone of IBD—including aminosalicylates, biologics, and immunomodulators—largely targets the inflammatory response. However, the challenges such as primary non-response, secondary loss of response, and systemic side effects are often problematic. Consequently, there is an urgent need to develop novel therapeutic and preventive strategies that target the underlying microbial and metabolic causes of the disease rather than modulating immune responses. This review integrates the pathomechanistic implications of the microbiome-metabolic axis in the maintenance of gut homeostasis and its disruption in IBD, with particular emphasis on the global epidemiology of the disease. We further evaluate emerging therapeutic and preventive strategies aimed at restoring the microbiome-metabolic axis, including fecal microbiota transplantation (FMT), probiotic therapy, bacteriophage therapy, and helminth-based therapies. In addition, we explore the potential of advanced approaches such as microbiome engineering and precision genome editing to enable highly personalized therapeutic paradigms. By bridging microbial ecology with clinical pathology, this review highlights the transformative potential of targeting the host-microbiota interface to achieve improved long-term outcomes in IBD.

## Introduction

Inflammatory bowel diseases (IBD), including ulcerative colitis (UC) and Crohn’s disease (CD), are chronic relapsing inflammatory conditions of the gastrointestinal tract that have imposed a considerable and increasing global health burden (Wang et al., 2023). Although UC and CD differ in their anatomical distribution and depth of inflammation, both are characterized by recurrent intestinal inflammation that impairs nutrient absorption along with their risk of complications. Despite decades of research, the precise pathogenesis of IBD is not completely understood, reflecting its multifactorial and complex nature (Jabłońska and Mrowiec, 2023).

Traditional understanding of IBD pathogenesis has centered on understanding the immune system, with a core emphasis on dysregulated and increased immune activation in response to luminal antigens (Zuo and Ng, 2018). However, this hypothesis alone is insufficient to account for the observed heterogeneity of disease, treatment responses, and patterns of recurrence (He et al., 2022). Increasing evidence supports the view that the IBD results from a complex and continuously shifting imbalance among host immunity, the gut microbiome and intestinal metabolism (Zheng et al., 2020). Patients having IBD commonly exhibit intestinal dysbiosis, generally characterized by reduced levels of beneficial Firmicutes and increased presence of opportunistic pathogenic microorganisms. Further, such changes are accompanied by functional alterations in microbial metabolism, including pathways involving short-chain fatty acids, bile acids, and redox homeostasis, which are the key players of mucosal inflammatory responses and immune system reprogramming (Hu et al., 2022). In addition, diet and nutrition are key factors influencing the interaction between gut microbes and the immune system. Dietary patterns influence the composition and metabolic activity of intestinal microorganisms, which in turn can shape the immune response in the mucosal lining of the intestine (Dawson et al., 2025). Disruption of the tripartite relationship between dietary habits, the gut microbes, and the immune system can contribute to the pathophysiology of IBD (Kou et al., 2025).

Although current therapeutic strategies including immunomodulators, aminosalicylates, biologics, and emerging small molecules are promising, concern remains regarding factors such as the durability of treatment responses and the adverse effects (Cai et al., 2021). Therefore, the need to develop alternative therapeutic approaches is crucial, focusing on the underlying causative mechanisms rather than modulation of immune function.

Within this context, the current review aims to discuss new therapeutic and preventive modalities that aim to rebalance the metabolic immune-microbiome axis in IBD. These include natural bioactive compounds, targeted probiotic interventions, fecal microbiota transplantation (FMT), bacteriophage-based therapies, and helminth-derived approaches, highlighting their mechanistic rationale and potential to reshape future IBD management.

## IBD prevalence: unraveling global epidemiology and susceptibility factors

IBD has emerged as a global health problem with increasing prevalence worldwide, especially in newly industrialized regions such as Asia, even though initially it was perceived as a predominantly Western disease (Aniwan et al., 2022; Caron et al., 2024). The increasing incidence of IBD globally and the differences in prevalence and severity of the disease between countries and regions specifically highlight the significance of identifying geographic variations and the type of IBD that predominates in a particular region (Zhou et al., 2023a). In 2019, approximately 4.9 million cases of IBD were reported worldwide, with the highest number of cases in China (911,405 cases; 66.9 per 100,000) and the United States (762,890 cases; 245.3 per 100,000). In the Asian population, the incidence is highest in the age group of 30–34 years, while the prevalence is highest in the age group of 45–49 years.

According to the recent Global Burden of Disease (GBD) 2021 analysis, approximately 3.83 million people were living with inflammatory bowel disease (IBD) worldwide, with 375,140 new cases and 42,423 deaths, resulting in 1.51 million disability-adjusted life years (DALYs). Furthermore, the epidemiological studies reveal a significant geographic shift in the distribution of inflammatory bowel diseases, where an instantaneous increase is now observed in emerging industrialized countries in Asia, South America, Eastern Europe, and Africa. The worldwide burden of IBD is significant, with a total of 7 million people suffering from the disease and a significant rise in the number of pediatric cases and adolescents, even in countries where IBD is rare, such as East Asia (Solitano et al., 2025).

In a recent large-scale study called “Global evolution of inflammatory bowel disease across epidemiologic stages,” researchers reviewed data collected from more than 500 population-based studies conducted across 82 regions for more than one hundred years and found that the development of IBD traverses through various epidemiologic stages, including emergence, accelerating incidence, compounding prevalence, and a potential future fourth stage that involves prevalence stability. Accordingly, industrialized areas are now progressing towards a stage of incidence stabilization, but prevalence rises due to demographic aging, while new industrialized regions are currently witnessing fast-rising incidences. While industrialized areas are observing a stabilization in the incidence of the condition, the prevalence is increasing due to an aging population. Conversely, newly industrialized regions are currently experiencing a rapid increase in incidence rates. Despite its comprehensive nature, there are certain limitations that are inherent in this study, which include heterogeneity of sources, different diagnostic standards, and projection modeling (Hracs et al., 2025).

These epidemiological variations underscore the complex relationship between environmental factors related to industrialization and the worldwide burden of inflammatory bowel diseases (IBD) making the disease inextricably related to a broad array of environmental factors, such as infectious agents, tobacco, drugs, psychological stress, atmospheric and aquatic pollutants, specific dietary habits, and food additives.

Quantitative evidence indicates that smoking increases the risk of Crohn’s disease by approximately 2.0-fold, while the ulcerative colitis risk increases by nearly 1.2-fold. Early-life antibiotic exposure is associated with a 2.0-fold higher risk of IBD development, and adherence to a Western dietary pattern confers nearly a 2-fold increased risk. However, breastfeeding strikingly reduced concomitant risk factors by 20–25%, and Mediterranean dietary adherence lowered the risk of progression by 20–30% (Chhibba et al., 2025).

Resonating the recent observations, the increase in IBD incidence and prevalence over the past 30 years has been most pronounced in regions with high socio-demographic indices, though a plateauing or even decreasing trend has been observed in North America, while Asia and parts of Africa, Latin America, and the Middle East continue to show increasing trends (Desai and Dhoble, 2024). This shift highlights the critical influence of socioeconomic development and lifestyle changes on disease incidence, necessitating comparative analysis of disease burdens across diverse geographical and economic contexts (Chen et al., 2025). The recent epidemiological framework proposes that early industrialized countries are currently in a compounding prevalence phase and are expected to transition toward prevalence equilibrium within the next decade, with more than 1% of their populations projected to live with IBD. In contrast, newly industrialized countries are anticipated to shift from accelerating incidence to compounding prevalence over the next 20 years, sustaining the global healthcare burden despite stabilizing incidence in high-income regions (Kaplan, 2025).

Sex is another biological variable that has been overlooked in genome-wide association studies (GWAS) of complex traits. In 2021, women accounted for about 2 million total cases and 22,968 deaths globally, whereas men had slightly more incident cases (188,005), underscoring sex-specific epidemiological patterns (Yang et al., 2020). Crohn’s disease (CD) is more commonly reported in females, whereas males diagnosed with IBD more often exhibit upper gastrointestinal tract involvement and ileal-predominant CD. On the other hand, in female cohorts with IBD, colonic diseases and extraintestinal manifestations have been found to be more prevalent. The above observations have been supported by a recent meta-analysis involving 17 cohorts in population-based studies regarding sex disparities in IBD (Khrom et al., 2023).

**Figure 1:**
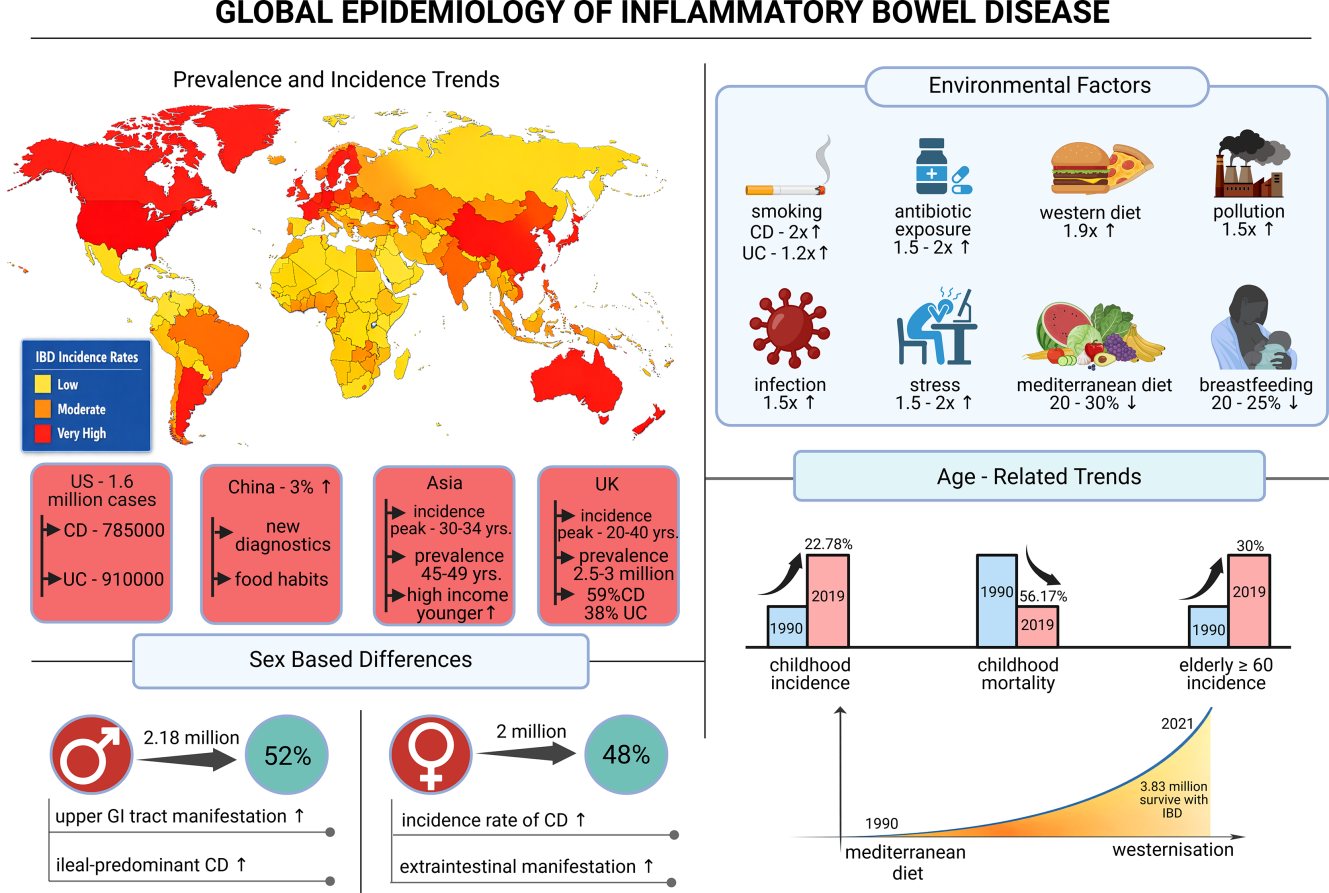
Global epidemiology and environmental determinants of IBD, inflammatory bowel disease. The world map represents the geographic epidemiology of IBD, highlighting high disease burden in North America and Europe and rapidly increasing incidences in Asia and other industrialized regions. Various environmental factors, such as smoking, antibiotic use, western dietary habits, infections, psychological stress, pollution, and reduced breastfeeding, increase the chance of IBD, while the Mediterranean diet has shown a protective effect. The age-related graph demonstrates an increasing incidence of IBD in pediatric populations and reduced mortality rates in them. Trends also depicted prolonged disease burden in older people. Sex-based differences are also illustrated, including a relative male predominance and variations in disease phenotype.

Another important demographic factor in relation to IBD prevalence is age, where there has been a notable trend in the onset of IBD at younger ages. According to data from the Global Burden of Disease (GBD) study conducted in 204 countries between 1990 and 2019, the incidence of IBD has increased by 22.78% in childhood cohorts worldwide, despite a 56.17% decrease in IBD mortality. The demographic landscape of IBD is shifting, with older patients now constituting the largest proportion of the IBD population, as evidenced by a rise in IBD patients aged ≥60 years from 20% to currently 30% (Caron et al., 2024). A schematic representation of the global trends in IBD prevalence is represented in **Figure 1**.

## Dysbiosis crossroads: pathobionts, commensals, and the inflammatory imbalance in IBD

IBD is often cross-linked to an imbalance, or dysbiosis, in the gut microbiome (Wlodarska et al., 2015). One key microbe implicated in this process is adherent-invasive *E. coli* (AIEC), a pathogen thought to contribute to both CD and UC (Nadalian et al., 2021). A characteristic histological feature of Crohn’s disease (CD) is epithelioid granulomatous inflammation within the intestinal tissue (Hong et al., 2020). Additionally, *Mycobacterium avium paratuberculosis* (MAP) has been proposed as a causative agent for Crohn’s disease. MAP has been isolated from the peripheral blood of patients with CD more frequently than from healthy individuals, although its pathological role remains a subject of ongoing investigation. Enteric pathogens like *Salmonella* spp., *Yersinia* spp., and *Shigella* spp., or the toxins they produce, are also implicated as potential contributors to UC pathogenesis (Doshi et al., 2024; Hendrickx et al., 2024; Lin et al., 2025). Furthermore, *Escherichia coli* strain p19A, isolated from the ileal mucosa of patients with CD and UC, has been shown to secrete α-hemolysin, which induces apoptosis in dendritic cells and promotes the expression of pro-inflammatory cytokines, including IL-6, IL-23, and TNF-α (Yang et al., 2020).

The gut microbiota of individuals with IBD also showed a characteristic compositional shift with a greater prevalence of phyla like Bacteroidetes and Proteobacteria (with increased abundance of *E. coli*), while there was a notable reduction in the phylum Firmicutes genera, such as *Lactobacillus*, compared to healthy controls (Salimi et al., 2022; Tsai et al., 2025).

Along with this, recent studies on patients with UC and CD highlight a significant reduction in anti-inflammatory bacteria such as *Roseburia* and *Phascolarctobacterium* spp (Liu et al., 2021a). This decline is directly associated with a substantial reduction in the production of SCFAs, particularly propionic acid, which are crucial for their anti-inflammatory properties. SCFA-producing bacteria, including *Faecalibacterium prausnitzii* (a key member of the *Clostridium leptum* group), are also markedly diminished in individuals with IBD. This reduction in beneficial bacteria coincides with an increased presence of certain *Clostridium* species, contributing to the overall dysbiosis observed in the gut microbiota of these patients (Haneishi et al., 2023).

Interestingly, certain microbes such as Bacteroides *vulgatus* have been reported to enhance epithelial barrier integrity, highlighting the complex and context-dependent roles of gut microbiota in IBD (Miller, 2024). *Helicobacter pylori*, a prominent inhabitant of the human gastric microbiota, has consistently shown an inverse correlation between the prevalence of *H. pylori* infection and the incidence of IBD. These findings suggest a potential protective role of the bacterium against the development of IBD (Imawana et al., 2020). This may involve modulating the host’s immune response shifting it away from a pro-inflammatory Th1/Th17 phenotype towards a regulatory T-cell response (Balani et al., 2023). Additionally, *H. pylori* infection can upregulate the production of antimicrobial peptides, which could suppress pathogenic bacteria implicated in IBD pathogenesis (He et al., 2024).

Recent studies in pediatric UC showed lower baseline gut bacterial diversity characterized by fewer butyrate-producing bacteria and an increase in oral-associated bacteria like *Veillonella parvula*, which is strongly associated with future relapse, with microbiota composition showing the highest predictive value, followed by host epigenomic and transcriptomic profiles. A machine learning model integrating microbial and epigenomic data could more accurately predict relapse than individual omics approaches, highlighting the prognostic potential of host-microbe interactions and suggesting that pro-inflammatory oral bacteria exploit reduced colonic diversity to drive disease recurrence (Kulecka et al., 2025).

At the epithelial interface, there is growing evidence that the process of restoring barrier integrity represents a key mechanistic node that integrates immune modulation, microbial function, and disease resolution in IBD. Pharmacological stimulation of epithelial repair mechanisms has been demonstrated to inhibit inflammatory signaling while maintaining tight junction structure. In this regard, the AhR-NR4A1 signaling pathway represents a fundamental mechanism that promotes epithelial homeostasis, in which downstream transcriptional upregulation of claudin-4, occludin, and ZO-1 promotes junction formation and inhibits paracellular permeability (Mao et al., 2025). The certain barrier-focused mechanisms are operative in tandem with dietary therapies such as exclusive enteral nutrition (EEN), which promotes remission through the reprogramming of microbial metabolic function rather than simply modulating microbial composition. EEN inhibits pro-inflammatory microbial pathways involving oxidative stress, including phenazine and indole diterpene alkaloid production, while activating energy metabolism and sphingolipid pathways that promote epithelial membrane integrity and signaling (Zheng et al., 2025). These observations collectively point to a unified mechanism by which epithelial repair and metabolic reprogramming inhibit inflammatory cascades and promote mucosal barrier integrity in Crohn’s disease.

In addition to epithelial dysfunction, the role of microbe-mediated immune signaling is critical in determining disease progression in IBD. Adherent-invasive Escherichia coli (AIEC) utilize unique virulence factors to overcome mucosal barriers and sustain chronic inflammation, such as LPF-mediated translocation at M cells in Peyer’s patches, connecting bacterial colonization to the early immune response in genetically predisposed individuals with NOD2 deficiency (Chassaing et al., 2011). In parallel, AIEC-secreted yersiniabactin disrupts macrophage metal homeostasis by sequestering intracellular zinc, blocking zinc-mediated HIF-1α hydroxylation, and consequently stabilizing nuclear HIF-1α to initiate a fibrotic transcriptional program that promotes intestinal fibrosis (Ahn et al., 2025).

## Reprogramming gut inflammation: diet as a therapeutic signal in IBD

The diagnosis and management of IBD necessitate a multidisciplinary approach, integrating the expertise of clinical gastroenterologists, endoscopists, radiologists, surgeons, pathologists, and clinical nutritionists (Rio et al., 2023). The multifactorial etiology of IBD, involving the gut microbiota, environmental factors, genetic predisposition, and personal behaviors such as diet and smoking, results in significant heterogeneity in its pathogenesis, mediated by epigenetic and immunological changes. This inherent heterogeneity presents a major obstacle to the successful development of effective therapeutics (Wan et al., 2025). Combining biologics with distinct mechanisms of action may invoke synergistic effects, thereby contributing to the management of refractory IBD (Xu et al., 2022). New treatment goals focus on achieving and maintaining deep remission, defined by mucosal healing, normalization of inflammatory biomarkers, and symptom resolution (Wetwittayakhlang et al., 2021).

Although the precise pathogenesis of IBD remains uncertain, a key pathological mechanism is a disruption in the balance between pro- and anti-inflammatory signaling. The pro-inflammatory cytokine tumor necrosis factor-alpha (TNF-α) represents the most extensively investigated inflammatory pathway in IBD (Zeng et al., 2024). Recent research has identified other potential molecular targets, including Janus kinase (JAK) inhibitors and anti-integrin adhesion molecules, for the management of acute inflammation (Furfaro et al., 2022). Given the limited efficacy of current biological monotherapies in a significant subset of patients, there is a substantial focus on the rapid development of new drugs to meet the diverse needs of the IBD patient population (Rogler et al., 2021).

Among modifiable environmental factors, diet has emerged as a critical regulator of intestinal inflammation through its ability to shape microbial composition, metabolic activity, and immune signaling. Dietary patterns influence epithelial barrier integrity, microbial metabolite production, and the balance between pro- and anti-inflammatory immune responses, thereby playing a central role in disease onset, progression, and remission. Increasing evidence suggests that targeted nutritional interventions can reprogram dysregulated immune responses indirectly via microbiome modulation, positioning diet as a promising adjunctive strategy for achieving sustained mucosal healing and long-term disease control in IBD.

The prospective DELECTABLE study demonstrated the feasibility of implementing the Crohn’s Disease Exclusion Diet (CDED), the whole-food additive-free diet (WFD), and the low-sulphur plant-based diet in clinical practice, with high patient adherence and satisfaction over 12 weeks (Trakman et al., 2025). The reduction in processed food additives by WFD was associated with a significant reduction in intake (p = 0.009), and both CDED and WFD resulted in a significant improvement in quality of life (p < 0.001). In patients with CD, CDED resulted in a significant reduction in objective inflammatory indices, such as C-reactive protein and fecal calprotectin, as well as CD Activity Index scores (p s< 0.045), whereas WFD resulted in a reduction in CDAI and partial Mayo scores in ulcerative colitis (p < 0.027). In light of these findings, a randomized controlled trial evaluating a Mediterranean and low FODMAP diet combination with partial enteral nutrition in active UC showed a significant reduction in disease activity index compared with baseline (p < 0.001) and with the control diet (p = 0.043). Marked improvements in quality of life (p < 0.001) and a significant reduction in high-sensitivity CRP (p < 0.01) were observed, underscoring the anti-inflammatory role of plant-based diets with carefully managed fermentable content (Narimani et al., 2024).

In addition to luminal disease management, preoperative nutritional optimization has been proved feasible and safe. A multicenter randomized trial demonstrated high levels of adherence to six-week regimens of exclusive enteral nutrition or CDED before elective Crohn’s surgery, with low rates of short-term postoperative complications (three Clavien-Dindo Grade II events at 30 days), suggesting the role of targeted nutritional therapy in the inflammatory modulation of the perioperative period (Wall et al., 2024). However, despite promising results with exclusive enteral nutrition, exclusion diets, Mediterranean diets, and low FODMAP diets, no dietary pattern has yet reached a universally accepted level of consensus for the long-term maintenance of remission, and unattended restrictive diets may place patients at risk for protein-energy malnutrition and deficiencies of iron, vitamin D, vitamin B12, and folate (Istratescu et al., 2024). Taken together, the results suggest that specific dietary practices focusing on organized and balanced nutritional patterns can downregulate inflammation and improve quality of life. However, these patterns work best when individualized in a multidisciplinary setting to maximize therapeutic benefit while ensuring nutritional safety.

## Alternative and complementary interventions: probiotic precision remodeling the gut microbiome

Therapeutic strategies for ulcerative colitis (UC) and Crohn’s disease (CD) are designed to mitigate inflammatory processes during disease flares, induce remission, and prevent long-term complications. Conventional pharmacological interventions include aminosalicylates, corticosteroids, and thiopurines. Certain drugs are highly effective class of drugs for inducing remission in IBD. However, chronic usage of certain therapeutics can lead to significant risks, including cardiovascular disease, cataracts, osteonecrosis, and gastrointestinal hemorrhage (Bruscoli et al., 2021; Koshi et al., 2022). These significant adverse effects and limitations of conventional IBD therapies have spurred a demand for alternative treatment modalities. Given the established role of gut microbiota dysbiosis in IBD pathogenesis, probiotics have emerged as a promising therapeutic strategy. These beneficial live microorganisms can re-establish a healthy microbial balance by introducing non-pathogenic bacterial or yeast strains, thereby modulating the intestinal microbiota and conferring a health benefit in both humans and animals (Santos et al., 2024). Recent research has incorporated the use of profiling the microbiome along with clinical data to categorize IBD patients into groups according to their microbial profiles and, therefore, design more effective probiotic treatments for each group. The underlying mechanism for this strategy is based on the interaction between the host and microbiome metabolism. In this case, metabolites like butyrate act on HDACs and induce the expression of Treg cells as well as inhibit inflammation through NF-κB-mediated signaling pathways. On the other hand, indole metabolites resulting from tryptophan metabolism act on the AhR to improve epithelial regeneration and mucosal immune tolerance (Modoux et al., 2022; Parada-Venegas et al., 2025).

**Figure 2:**
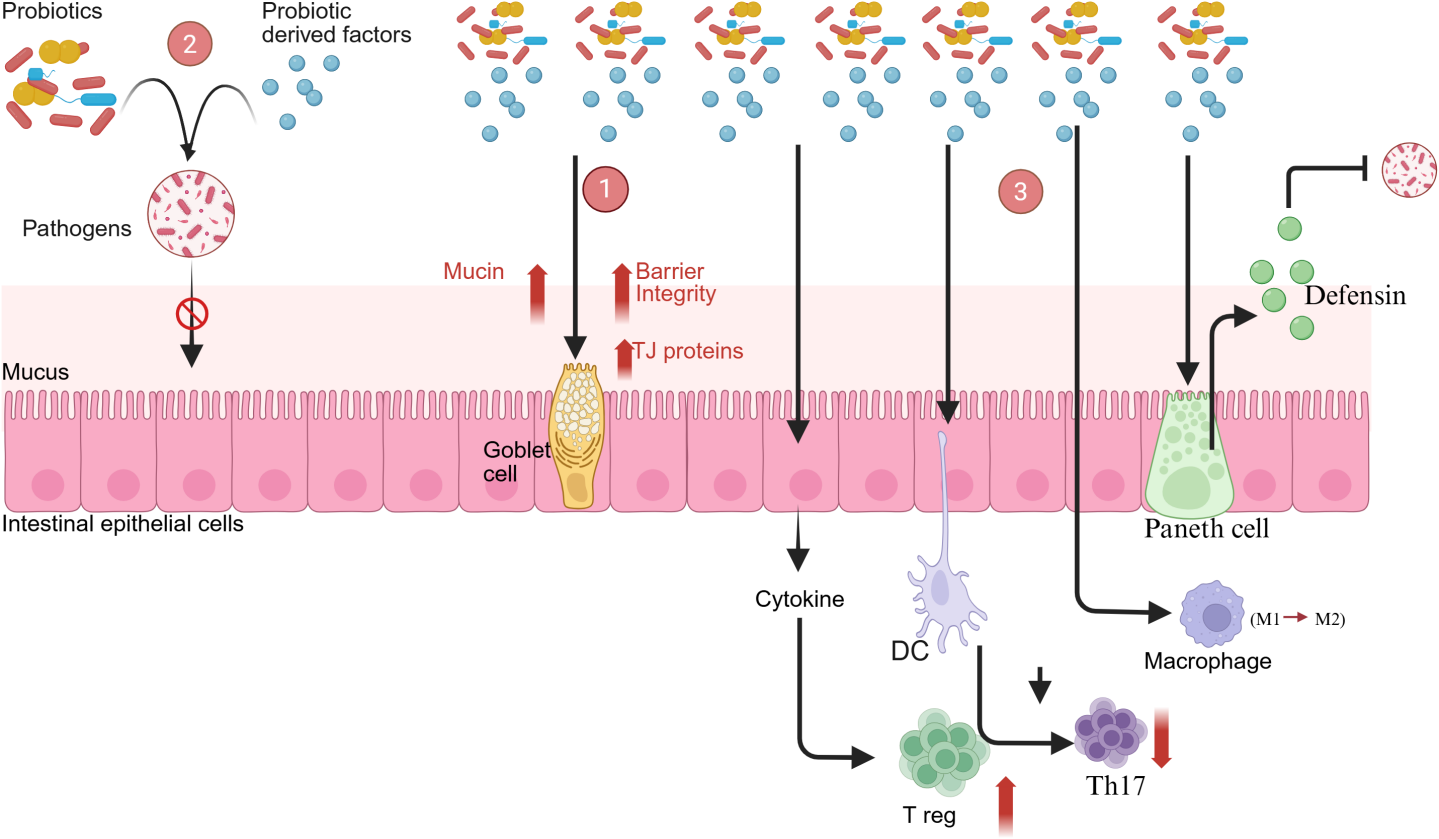
Mechanism of probiotic-mediated effects on the intestinal epithelium.

*Saccharomyces boulardii*, *lactobacilli*, and *bifidobacteria* strains are clinically available probiotics used to manage gastrointestinal infections (Anjana and Tiwari, 2022). A key functional characteristic of these microorganisms is the production of bacteriocins, and the therapeutic benefits include modulation of the intestinal microbiota, stimulation of host immunity, enhancement of nutrient bioavailability, and reduction in the symptoms of lactose intolerance (Latif et al., 2023). A summary of the mode of action of probiotics is represented in **Figure 2**. Probiotics have been shown to modulate the inflammatory cytokine profile in IBD patients, leading to a reduction in pro-inflammatory markers like TNF-α, IL-8, and IFN-γ in the serum. Concurrently, probiotic administration elevates the levels of anti-inflammatory cytokines, specifically TGF-β and IL-10. This dual action underscores the role of probiotics in restoring immunological homeostasis within the gut. These beneficial effects are mediated by several mechanisms, including the enhancement of intestinal barrier function and the regulation of key host cell signaling pathways such as Akt, NF-κB, and mitogen-activated protein kinases (MAPKs). These actions collectively promote the survival of intestinal epithelial cells and regulate immune responses.

**Figure 3:**
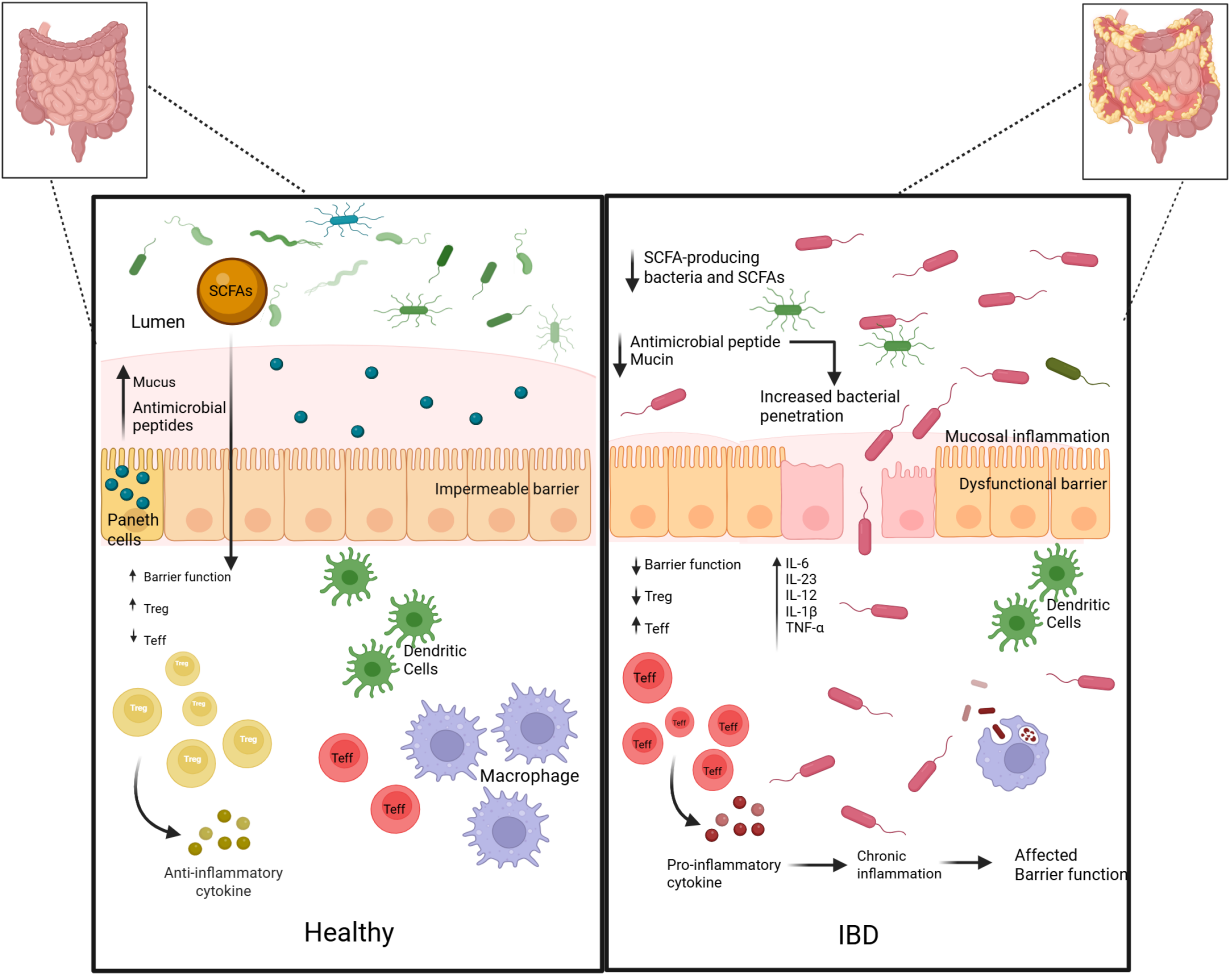
Implications of a healthy gut in comparison with an IBD-induced dysfunctional gut barrier. The left panel represents healthy homeostasis in the gut, where a robust impermeable barrier is maintained by a dense mucus layer with exacerbated expression of antimicrobial peptides and mucins secreted by Paneth and goblet cells. Microbial-derived metabolites, particularly SCFAs, short-chain fatty acids, penetrate the epithelial layer to promote Treg, T regulatory cell differentiation and the secretion of anti-inflammatory cytokines, maintaining immune tolerance. The right panel represents IBD-induced dysbiosis, leading to a significant reduction in SCFA-producing bacteria and protective mucosal factors, implicating a dysfunctional, leaky epithelial barrier. This promotes increased bacterial penetration and the subsequent activation of dendritic cells and macrophages. The resulting pro-inflammatory cascade characterized by elevated levels of IL-6, IL-23, IL-12, IL-1β and TNF-ɑ drives the expansion of Teff, effector T cells perpetuating chronic inflammation compromising epithelial integrity.

The antimicrobial peptides produced by the probiotic strain serve to enhance the barrier function of the epithelium by killing pathogenic bacteria (Gubatan et al., 2021) (**Figure 3**). Probiotics also exhibit direct impacts on barrier integrity via modulation of tight junctions via the AMPK and PI3K/Akt pathways, resulting in increased levels of occludin, claudin-1, and ZO-1. ZO-1 phosphorylation thereby restores its assembly with claudins and occludins (tight junction components), thereby improving epithelial barrier integrity. Moreover, strains like *Lactobacillus rhamnosus* GG increase mucin (MUC2) secretion and goblet cell maturation, improving the quality of the mucosal layer and reducing translocation of bacteria (Park et al., 2022). Clinically, reduced barrier function is associated with disease recurrence and severity in IBD, underscoring the importance of re-establishing epithelial integrity as a target of treatment (Poritz et al., 2011; Ferris et al., 2025; Yang et al., 2023). Other notable probiotic strains include *Akkermansia muciniphila, a* key commensal bacterium effective in restoring eubiosis in chronic colitis, and *Lactobacillus reuteri*, which produces the broad-spectrum antimicrobial compound reuterin (Zhang et al., 2024a). Furthermore, *Lactobacillus casei* exerts its immunomodulatory effects by downregulating IL-6 and TLR-3 expression while also fortifying the host epithelial barrier via the enhancement of goblet cell function. Probiotics, their derived postbiotics, and mode of action are depicted in **Table 1**.

**Table 1:** Postbiotics and their mode of action in IBD.

Strains	Derived postbiotics	Functions
*Bifidobacterium longum* CECT-7347	Dead cell (heat-killed)	Anti-inflammatory activity – reducing acute inflammatory response (Srivastava et al., 2024).
*Bifidobacterium longum* 35624	Exo-polysaccharide (EPS)	Anti-inflammatory activity – NF-κB activation and cytokine secretion modulation (Schiavi et al., 2016).
*Lactobacillus sp.*	EPS	Anti-inflammatory activity, Increase in IL-10 and decrease in TNF-α (Zhang et al., 2024b).
*Lactobacillus paracasei* D3–5	Lipoteichoic acid	Anti-inflammatory activity (Enhancing Muc2 expression by modulating TLR-2/p38-MAPK/NF-kB pathway) (Zhang et al., 2024b).
*Lactobacillus rhamnosus* GG	Bacterial lysates	Protects intestinal epithelial integrity by enhancing barrier function, reducing mucosal inflammation, and preventing bacterial translocation that contributes to colitis and intestinal permeability (Park et al., 2022).
*L*. *acidophilus*	Peptidoglycan	Proinflammatory reduction (Islam et al., 2024).
*L*. *plantarum* *L*. *paracasei* *L*. *rhamnosus*	Teichoic acids	Anti-bacterial and Anti-inflammatory activity (Islam et al., 2024).
*Lactobacillus sp.* *Lactococcus lactis* *S. thermophilus* *L*. *casei* *L*. *fermentum*	Enzymes	Reduction in intestinal inflammation (Islam et al., 2024).

In a complementary manner, prebiotics, such as fructooligosaccharides (FOS) and galactooligosaccharides (GOS), selectively promote the growth of beneficial commensal bacteria, including *Bifidobacterium* and other Firmicutes. Specifically, GOS has been linked to a reduction in harmful bacteria like *Desulfovibrio* and an increase in *Bifidobacterium*, which may lead to decreased hydrogen sulphide levels and enhanced butyrate production. Other prebiotic compounds, including germinated barley foodstuff (GBF), psyllium, lactulose, and the non-saccharide metabolite 1,4-dihydroxy-2-naphthoic acid, are also being investigated for their therapeutic potential in ulcerative colitis (Kennedy et al., 2024).

Genetically engineered probiotics represent a novel and targeted delivery platform for therapeutic molecules to the gut mucosa, which can overcome the systemic side effects and high costs associated with conventional therapies. A systematic review of twelve preclinical studies demonstrated that genetically modified probiotics engineered to produce the anti-inflammatory cytokine IL-10 exhibited superior efficacy in attenuating intestinal inflammation and tissue pathology compared to their non-recombinant counterparts. These studies reported a significant amelioration of clinical disease indices and a reduction in colon shortening. Immunological profiling consistently showed a marked upregulation of IL-10 expression, which correlated with a downregulation of key pro-inflammatory cytokines such as IFN-γ, TNF-α, and IL-6. This effect was particularly pronounced when utilizing engineered strains of *Bifidobacterium longum*, highlighting the potential for strain-specific therapeutic applications. The clinical viability of this approach was further supported by a small-scale clinical trial where a recombinant *Lactococcus lactis* strain engineered to produce IL-10 (LL-Thy12) demonstrated both safety and a reduction in clinical disease activity in patients with CD. Beyond IL-10, other recombinant probiotics have shown promise; for instance, *L. lactis* engineered for IL-27 production (LL-IL-27) and other recombinant probiotics expressing IL-35 have shown enhanced efficacy in preclinical colitis models. These therapeutic effects are mediated by the attenuation of intestinal pathology, improvement of disease indices, and beneficial modulation of the cytokine milieu (Zhang et al., 2023).

While these data underscore the effectiveness of engineered probiotics, they simultaneously generate biosafety concerns, especially for vulnerable and immunocompromised patients (Wang et al., 2026). One of the major risk factors is the horizontal gene transfer (HGT), which can spread antibiotic resistance or virulence characteristics, leading to the emergence of superbugs. Translational challenges such as variability in patient responses, lack of survival during gastrointestinal passage, and limitations regarding colonization also raise more concerns. Overall, the lack of standardized regulatory frameworks and sufficient data regarding long-term safety highlights the urgent need for rigorous evaluation and controlled clinical translation of recombinant probiotics (Spacova et al., 2023; Duan et al., 2025).

Additionally, the increasing integration of engineered probiotic strains into clinical practice brings forth notable ethical implications. They are primarily focused on patient safety, biosafety, and ecological sustainability in the long term. These risks are further exacerbated by the current limitations of preclinical models, as experimental models cannot possibly capture the complexity of the human gut microbiome, thus necessitating careful clinical translation with rigorous regulatory supervision (Wei et al., 2023; Duan et al., 2025). The need for accurate strain identification, comprehensive genomic characterization, and adverse event reporting has also been highlighted to encourage transparency and scientific integrity (Merenstein et al., 2023). The occurrence of severe adverse events such as sepsis, bacteremia, and fungemia in susceptible patients further illustrates that live microbial therapies cannot be considered universally safe and that there is an ethical imperative to perform rigorous safety assessments and explore safer alternatives such as postbiotics (Pandey et al., 2025).

(1) Probiotic-derived factors promote goblet cells and upregulate tight-junction proteins (OCLN, TJP1), thereby enhancing epithelial barrier integrity. (2) Secreted metabolites and cell-free supernatants (CFS) disrupt quorum sensing (QS) and biofilm matrices, inhibiting pathogen adhesion to the intestinal epithelium. (3) Immunomodulatory signaling through C-type lectin and CARD9 pathways activates antimicrobial peptide (AMP) secretion from Paneth cells, promotes M2 macrophage polarization, and enhances T regulatory (Treg) cell activity to downregulate infection-driven inflammatory responses.

## SCFA symphony: orchestrating anti-inflammatory harmony in the inflamed gut

Intestinal microorganisms metabolize dietary fibers to produce the major short-chain fatty acids (SCFAs): acetate, propionate, and butyrate, which are present in the colon at a ratio of approximately 3:1:1, respectively. These immunomodulatory compounds possess significant properties, supporting the proliferation and function of various immune cell populations. In patients with IBD, there was a noticed decrease in the abundance of primary SCFA-producing bacteria, such as *Faecalibacterium prausnitzii* and *Roseburia intestinalis*. This microbial dysbiosis correlates with significantly reduced luminal SCFA concentrations when compared to healthy controls (Parada Venegas et al., 2019; Nogal et al., 2021).

SCFAs modulate their functions primarily through G protein-coupled receptors (GPR43/FFAR2, GPR41/FFAR3, GPR109A, and PSGR), which are widely expressed in intestinal epithelial and immune cells and actively regulate hormone secretion, lipid metabolism, and anti-inflammatory responses through these receptors. One of the primary roles of SCFAs is to maintain the integrity of the epithelial barrier by modulating tight junction dynamics. The SCFAs, especially butyrate, bind to the receptors like GPCRs that lead to activation of subsequent signaling cascades, including PKCβ and AMPK pathways. The phosphorylation of the occludin and ZO-1 mediated via PKC causes their localization in apical membranes, and AMPK further increases the binding of ZO-1 to F-actin. Structurally, claudin and occludin control the paracellular transport, and ZO-1 associates them with the actomyosin cytoskeleton (Pérez-Reytor et al., 2021; Liu et al., 2024).

Additionally, butyrate, propionate, and acetate could express anti-inflammatory responses by counteracting the upregulation of different cytokines mediated by LPS-induced immunological reactions. LPS can activate TLR pathways further, leading to the activation of NF-κB and MAPK pathways, upregulating pro-inflammatory cytokines such as TNF-α, IL-1β, and IL-6. This action is countered by SCFAs through mechanisms involving G protein-coupled receptors and histone deacetylase (HDAC) inhibition, further increasing the production of anti-inflammatory cytokines such as IL-10. Additionally, SCFAs modulate inflammatory responses of macrophages by downregulating the expression of IL-8 (He et al., 2020).

Furthermore, butyrate promotes anti-inflammatory effects by inducing the differentiation of regulatory T cells (Tregs), which are crucial for modulating and suppressing excessive immune responses (Firoozi et al., 2025). Propionate, another key short-chain fatty acid, exerts its immunomodulatory effects by specifically binding to the G-protein-coupled receptors GPR41 and GPR43, which are expressed on various immune cells. In the DSS-induced colitis model, the critical role of GPR43 was studied, as the GPR43-deficient mice showed increased severity of colonic inflammation compared to wild-type controls. This was evidenced by increased histological scores, neutrophil infiltration, and elevated levels of pro-inflammatory cytokines like TNF-α and IL-17. Furthermore, a high-fiber diet or direct GPR43 activation effectively suppressed colonic inflammation in both wild-type and germ-free mice, an effect that was completely abrogated in the GPR43 KO mice. These findings strongly suggest that GPR43 is essential for mediating the anti-inflammatory properties of SCFAs within the intestinal mucosa (Parada Venegas et al., 2019).

Moreover, a randomized study performed on patients with UC, supplemented with 600 mg/kg of sodium butyrate, has shown an effective decrease of disease severity, supported by a significant decrease in the partial Mayo score. Beyond clinical symptoms, the intervention effectively lowered systemic inflammatory markers, specifically erythrocyte sedimentation rate (ESR) and the neutrophil-to-lymphocyte ratio (NLR) (Firoozi et al., 2025). Furthermore, the anti-inflammatory nature of butyrate was evidenced by the suppression of cytokine secretion in gut mucosal tissue of IBD patients along with the reduction of pro-inflammatory markers in immune cells, which was mediated through HDAC3 inhibition, as evidenced by increased histone acetylation (H3K9ac) (Parada-Venegas et al., 2025). Clinical studies have indicated doses of 0.15–0.30 mg/kg of body weight for precise colonic propionate delivery, where they can function as powerful metabolic modulators in humans. Linking towards measurable downregulation of markers associated with systemic inflammation. This protective mechanism suggests that propionate acts not just as a metabolic fuel, but as a signaling molecule that preserves intestinal homeostasis (Chambers et al., 2015, 2019; Facchin et al., 2025).

Even though evidence has shown the effectiveness of SCFA in IBD patients, evident limitations include inconsistent and modest clinical outcomes. Translational studies have shown SCFA enemas inducing remission only in a subset of patients, with negligible or non-effective outcomes in various indicators, including symptom scores, stool consistency, oxidative stress, and mucosal healing. Similarly, in diversion colitis, SCFA treatment fails to produce significant endoscopic or histological changes. There is also a lack of understanding about cross-feeding mechanisms, optimal SCFA composition and dosing, and long-term therapeutic impact. Therefore, well-designed randomized controlled trials and diverse experimental models are needed to better define the clinical utility of SCFAs in IBD treatment (Parada Venegas et al., 2019).

## Antimicrobial peptides: innate defenders and diagnostic sentinels in IBD pathogenesis

Antimicrobial peptides (AMPs) are recognized as promising biomarkers and novel therapeutic targets in inflammatory bowel disease (IBD). Specifically, calprotectin and lactoferrin are established AMP-based biomarkers used for monitoring disease activity in patients with IBD. A prominent class of AMPs is the defensins, which protect against bacterial infection by directly disrupting microbial membranes. The α-defensins, including human defensin 5 (HD5) and human defensin 6 (HD6), are secreted by Paneth cells in the intestinal crypts. Notably, HD6 has been shown to provide a physical barrier against infection by blocking the invasion of intestinal epithelial cells. Furthermore, the expression of β-defensins like BD-2 and BD-3 is significantly increased in response to infectious stimuli, underscoring their critical role in the host’s innate immune response (Gubatan et al., 2021). Cathelicidin is a family of defense peptides, with the human variant known as LL-37 and the murine equivalent as mCRAMP (Yu et al., 2021). These peptides function by pore formation in bacterial cell membranes (Zhang et al., 2023). Beyond their direct antimicrobial action, they inhibit fibrogenic gene expression in fibroblasts and have been linked to the activation of the TLR9-ERK signaling pathway while also providing a protective role against colitis (Mohamed et al., 2020; Gubatan et al., 2021). Elafin is another low-molecular-weight antiproteinase that acts as an “alarm” protein, preventing excessive inflammation (Deraison et al., 2023). It’s locally expressed by epithelial and immune cells and can modulate the NF-κB pathway, cytokine secretion, and the recruitment of inflammatory cells (Krawiec and Pac-Kożuchowska, 2022). Lactoferrin, a glycoprotein, is a valuable biomarker for neutrophil activity. It can modulate the NF-κB pathway to reduce inflammation (Ohradanova-Repic et al., 2023). Fecal lactoferrin is particularly useful as a sensitive and specific marker for differentiating active from inactive IBD and for predicting disease relapse (Langhorst et al., 2020). Microcin J25 (MccJ25), a 21-amino-acid lasso peptide, belongs to the microcin family. It effectively eliminates gram-negative bacteria by inhibiting RNA polymerase (RNAP), a central enzyme in bacterial gene expression. Studies using a DSS-induced murine colitis model have demonstrated the anti-inflammatory properties of MccJ25, which significantly attenuates colitis-associated parameters, including the restoration of body weight loss, reduction in the disease activity index (DAI), and improvement in colon length (Shang et al., 2021).

In unicellular organisms, antimicrobial peptides (AMPs), particularly bacteriocins, function to suppress competing bacteria by disrupting their cell membranes. These compounds are thought to have a lower propensity for inducing resistance compared to conventional antibiotics (Moretta et al., 2021; Zhou et al., 2025). Colicin-like bacteriocins, with their narrow-spectrum activity, serve as an alternative approach to targeting harmful microorganisms by mechanisms such as creating membrane pores, acting as a nuclease, or disrupting peptidoglycan metabolism (Arbulu and Kjos, 2024). Studies in colitis models have established the role of colicins as key regulatory factors in the inflammation-induced proliferation (“bloom”) of *Enterobacteriaceae*. For instance, induced colicin production in commensal bacteria has been shown to be outcompeted and replaced by pathogenic *Salmonella* strains, highlighting the complex microbial interactions within the gut (Marković et al., 2022). These findings underscore the potential role of microbial interactions in gut health; however, further research could explore the specific anti-inflammatory effects of bacteriocins, either as independent entities or through their modulatory influence on the gut microbiota. For instance, Nisin F from *Lactococcus lactis* and thuricin CD from *Bacillus thuringiensis* have shown to stabilize the gut microbiota and selectively target pathogens like *Clostridioides difficile* without impacting commensal species. Similarly, LAB bacteriocins, such as pediocin PA-1 and enterocin CRL-35, are effective against foodborne pathogens like *Listeria monocytogenes*. Furthermore, some bacteriocins, including nisin Z and pediocin AcH, reduce pathogen colonization when administered preventively. The immune-stimulatory effects of nisin, such as increasing T lymphocyte counts, have also been noted, demonstrating a broader influence beyond direct antimicrobial activity (Anjana and Tiwari, 2022).

## Bioactive compounds in the quest for IBD treatment

Natural compounds and herbal products are gaining recognition for their potential to treat IBD by leveraging their rich content of phytoconstituents like catechins, flavonoids, terpenes, alkaloids, anthocyanins, quinones, and anthoxanthins (Duan et al., 2021). However, their efficacy in treatment depends upon certain factors, including compound type, bioavailability, and level of clinical evidence. Among phytochemicals, resveratrol and curcumin are two of the compounds extensively studied, with investigations spanning both preclinical and clinical studies. Resveratrol has demonstrated therapeutic efficacy in treating inflammatory bowel disease (IBD) by mitigating mucosal inflammation. It achieves this by downregulating pro-inflammatory mediators, including TNF-α, NF-κB, and ROS. Furthermore, resveratrol enhances the expression of superoxide dismutase (SOD), a key antioxidant enzyme, and can reduce the expression of toll-like receptor 4 (TLR4) on activated cells, further suppressing the inflammatory cascade (Li et al., 2022; Prakash et al., 2024).

Resveratrol bioavailability studies have shown its limited bioavailability due to the extensive phase II metabolism in the liver, where it is then converted into biologically active conjugates such as resveratrol-3-O-sulphate, resveratrol-3-O-glucuronide, and resveratrol-4′-O-glucuronide. Preclinical studies of resveratrol in animal models have shown reduction in histopathological indices, indicating its potential in improving tissue structure and attenuating inflammation (Ramírez-Garza et al., 2018; Gu et al., 2024).

Curcumin, on the other hand, primarily demonstrates an anti-inflammatory mechanism by suppressing pro-inflammatory transcription factors like NF-κB and the subsequent reduction of pro-inflammatory cytokines (Sharifi-Rad et al., 2020). Preclinical studies of curcumin treatment performed in Wistar rats have demonstrated reduction in myeloperoxidase (MPO) activity, TNF-α, and nitrite levels and downregulation of pro-inflammatory mediators such as COX-2 and iNOS, leading to the reduction of colonic damage and chronic inflammation (Camacho-Barquero et al., 2007). A meta-analysis of curcumin in a randomized controlled trial revealed that it significantly improved remission and clinical response in patients with ulcerative colitis (UC). However, when compared to placebo, curcumin did not demonstrate the same potential for remission in patients with Crohn’s disease (CD) (Mohseni et al., 2025).

Other phytochemicals, including *Aloe vera*, *Coriolus versicolor, Garciniacambogia*, ginger, and saffron, have also shown their anti-inflammatory effects primarily in preclinical studies by down-regulating the expression of pro-inflammatory mediators such as TNF-α, IL-6, and NF-κB. However, their translational impact remains limited and unconfirmed (Mao et al., 2019; Sánchez et al., 2020; Impellizzeri et al., 2022; Jing et al, 2022; Yang et al., 2023; H. Baky et al., 2022; Martínez-Burgos et al., 2022; Mir et al., 2024). Another set of traditional and emerging compounds, including *Ulmus rubra, Brahmi, Mume fructus, Lycium barbarum, and* isoflavones demonstrated anti-inflammatory nature, epithelial barrier function enhancement and modulation of gut microbiota (Ried et al., 2020; Wu et al., 2020; Ma et al., 2021; Martínez-Burgos et al., 2022; Zhou et al., 2023b; Roy et al., 2024). A summary of the bioactive properties of phytochemicals that can show potential in IBD treatment is represented in **Table 2**.

**Table 2:** Natural compounds and their mode of action in IBD.

Natural compound	Mode of action
*Curcuma longa* (Curcumin)	Suppresses NF-κB activation, leading to reduced transcription of pro-inflammatory cytokines and attenuation of immune-driven inflammation in IBD (Mohseni et al., 2025).
*Aloe Barbadensis Miller* (Aloe vera)	Suppresses colonic secretion of inflammatory mediators (prostaglandin E2, TNF-α, IL-8, and IL-12), resulting in anti-inflammatory effects, and exhibits antimicrobial activity that enhances its therapeutic potential in the colon (Sánchez et al., 2020).
*Coriolus versicolor* (cloud mushroom)	Suppresses the expression of pro-inflammatory mediators (TNF-α, NF-κB, IL-1β, IL-8, and IL-6), thereby modulating immune pathways and mitigating intestinal inflammation in IBD; additionally, it exerts anti-inflammatory and anti-neoplastic effects that enhance its therapeutic potential (Jędrzejewski et al., 2020, Impellizzeri et al., 2022; Jing et al., 2022; Yang et al., 2023).
*Ulmus Rubra* (slippery elm)	The bark exerts antioxidant activity that attenuates oxidative stress, a key contributor to IBD pathogenesis, thereby alleviating gastrointestinal inflammation (Joo, 2014).
*Bacopa monnieri* (Brahmi)	Enhances immunomodulatory responses and exerts anti-inflammatory effects, contributing to immune homeostasis in IBD (Roy et al., 2024).
*Garcinia cambogia* (Malabar tamarind)	Reduces levels of inflammatory mediators, including prostaglandin E2 and IL-1β, and inhibits myeloperoxidase expression, thereby decreasing oxidative stress and inflammation (Espirito Santo et al., 2020).
*Zingiber officinale* (Ginger)	Demonstrates cytotoxic and anti-inflammatory activities through downregulation of NF-κB signalling and suppression of IL-17 expression (Promdam and Panichayupakaranant, 2022).
*Crocus sativus* (saffron)	Attenuates inflammation in chemically induced colitis by downregulating pro-inflammatory cytokines, highlighting its therapeutic potential in IBD management (Mir et al., 2024).
*Lycium barbarum* (goji berries)	Reduces inflammatory cytokines, alleviating colitis symptoms; enhances tight junction protein expression to improve intestinal barrier function and modulates gut microbiota composition to support intestinal health (Vidović et al., 2022).
Isoflavones (from soybeans)	Inhibits NF-κB signaling, a central regulator of immune and inflammatory pathways, thereby suppressing inflammation in colitis (Wu et al., 2020).

However, research on natural compounds is largely limited to *in vitro* and animal experiments, which cannot completely recapitulate the complexity of human diseases. Moreover, there remains a lack of comprehensive understanding regarding the underlying mechanisms of action, as well as inadequate structural characterization of these compounds and their active constituents. Additionally, insufficient evaluation of toxicity, safety, pharmacokinetics, and pharmacodynamic profiles is an added concern. Emerging research focusing on natural compounds should prioritize well-designed clinical trials, in-depth mechanistic insights, and systematic safety assessments, along with exploring combination therapies to enhance therapeutic efficacy (Zhou et al., 2023b).

## Fecal microbiota transplantation: re-engineering the gut ecosystem for IBD resolution

Fecal microbiota transplantation (FMT), or fecal bacteriotherapy, restores the gut microbiota in IBD patients by transferring filtered stool from a healthy donor to the recipient’s gastrointestinal tract, thereby modifying the recipient’s microbial composition and improving health (Biazzo and Deidda, 2022). Over the previous decade, FMT has undergone a significant shift in perception, transitioning from a limited, evidence-deficient alternative therapy to a potentially effective first-line treatment option (Boicean et al., 2023). Studies suggested that pooled fecal material from multiple donors may be more effective than a single donor due to increased microbial diversity, a factor that has also made FMT a widely adopted and highly successful treatment for recurrent *Clostridioides difficile* infections (CDI) (Tan et al., 2020; Cha and Sonu, 2025). FMT received approval from the US Food and Drug Administration (FDA) in 2013 for clinical application in the management of recurrent or refractory *Clostridioides difficile* infection (CDI), a decision predicated on its documented efficacy, which has demonstrated success rates approaching 90% in specific instances.

As per the retrospective analysis done in 101 active UC patients undergoing multiple FMTs via colonoscopy, they have identified abdominal discomfort (30.8%), flatulence (15.9%), abdominal distension (9.8%), borborygmi (7.9%), and low-grade fever (7.6%) as the most frequent short-term adverse events. Long-term adverse events included arthritis/arthralgia (6.5%), urticaria (4.3%), depression (2.2%), allergic bronchitis (2.2%), and partial sensorineural hearing loss (2.2%). These findings suggest that FMT may be a well-tolerated and safe procedure in UC patients (Tan et al., 2020). The choice of administration route for fecal microbiota transplantation (FMT) is largely determined by the characteristics of the donor microbiota and the patient’s clinical condition. The lower gastrointestinal tract is the primary route, with fecal suspension delivered via colonoscopy or rectal enema (Cha and Sonu, 2025). Alternatively, FMT can be administered through colonoscopy, a nasogastric tube, a nasojejunal tube, or enemas, where the enemas are often recommended as a less invasive option for patients with severe CDI (Liu et al., 2021b; Gulati et al., 2023).

Oral fecal microbiota capsules are considered one of the effective and convenient options for treating recurrent *Clostridioides difficile* infection (CDI) compared to other routes of administration (Du et al., 2021). A meta-analysis of four randomized controlled trials in ulcerative colitis (UC) demonstrated significantly higher remission rates with fecal microbiota transplantation (FMT), with 28% (39/140) of patients achieving remission compared to 9% (13/137) in the placebo group. A larger systematic review and meta-analysis of 27 studies comprising 596 adult and pediatric IBD patients further reported a clinical remission rate of 28.8% (132/459) and an overall clinical response rate of 53% (241/459), with remission being more pronounced in adult UC cohorts (Tan et al., 2020). Similarly, in Crohn’s disease, a study of 174 patients found a remission rate of 20.1% and a response rate of 43.7% following FMT via mid-gut routes such as nasojejunal tube, endoscopy, and terminal ileum administration. These collective findings highlight the safety, tolerability, and therapeutic potential of FMT in IBD, particularly in adult populations (Xiang et al., 2020).

FMT therapy is generally considered safe; still, some of the clinical studies have shown mild adverse events post-therapy, including borborygmus, increased stool frequency, vomiting, transient fever, mild diarrhea, abdominal cramping, bloating, fatigue, nausea, and rectal discomfort. Some patients have also experienced headaches and sore throats, particularly in the case of nasogastric administration. In most of the cases, certain symptoms have been resolved within 2 days after treatment (Sbahi and Palma, 2016).

Various factors can influence the outcome of FMT therapy in IBD patients. It encompasses several variables, including the differences in the patient populations, disease severity, donor and recipient gut microbiota, host immune responses, genetic background, and post-transplant follow-up. Aspects related to the therapeutic strategies, including stool volume, preparation of FMT composition, number of infusions, route of administration, and use of adjuvant therapies, further contribute to treatment outcomes (Sbahi and Palma, 2016; Tian et al., 2024a). FMT-based remission reports and endoscopic evaluations have also shown variabilities ranging from very low to very high evidence rates. Furthermore, variations in study design, including sizes and inclusion of randomized control trials and observational studies, contribute to heterogeneity in results. In general, the variability in both efficacy outcomes and evidence quality limits definitive conclusions regarding the effectiveness of FMT in IBD (Malik et al., 2025).

## From viral dysbiosis to targeted immunomodulation: phages, eukaryotic viruses, and helminthic strategies in IBD

The gut virome, dominated by bacteriophages, is crucial in the maintenance of gut homeostasis through the regulation of bacteria and their interaction with the immune system (Cao et al., 2022). This regulation is achieved through bacteria-specific predation and the modulation of the bacteria dynamics, leading to the regulation of the release of cytokines and the mucosal immune response (Emencheta et al., 2023). Apart from bacteriophages, the role of eukaryotic viruses in the development of IBD has been increasingly recognized, especially through their interaction with loci linked to IBD (Tian et al., 2024b). This is achieved through the interaction of the virome with the pattern recognition receptors, including the Toll-like receptors, which regulate the innate and adaptive response to the virome (Jansen and Matthijnssens, 2023). Genome-wide association studies have identified the association of the loss of function in the IFIH1 gene, which encodes the MDA5 protein, and the susceptibility to IBD. Moreover, the profiling of the virome has demonstrated the increased levels of bacteriophage and eukaryotic viral sequences in the gut of CD patients, with bacteriophages being the distinguishing feature in CD and UC (Adiliaghdam et al., 2022). Specific alterations include increased phages targeting *Faecalibacterium prausnitzii*, enrichment of Caudovirales, Picornaviridae, and Anelloviridae, and reduced Microviridae abundance (Wu et al., 2025).

Environmental factors such as antibiotics, diet, radiation, and nitric oxide can trigger prophage induction, facilitating horizontal transfer of antimicrobial resistance and virulence determinants (El Haddad et al., 2022). Elevated levels of Enterovirus B in intestinal tissues and increased Epstein–Barr virus DNA in peripheral blood mononuclear cells further underscore virome perturbations in IBD (Axelrad et al., 2021). Additionally, polymorphisms in FUT2 and FUT3, which regulate histo-blood group antigen expression, have been linked to altered viral interactions and increased disease susceptibility. Although strong associations between virome alterations and IBD phenotypes are evident, causal relationships remain incompletely defined, necessitating functional studies to determine whether virome changes act as drivers, amplifiers, or consequences of inflammation (Tarris et al., 2021).

Based on these mechanistic observations, and in contrast to more clinically established microbiome-targeted approaches such as dietary interventions and fecal microbiota transplantation (FMT), bacteriophage-based therapy is being developed as a targeted but relatively early-stage therapeutic option. Bacteriophages have been highlighted for their therapeutic potential through strain-specificity and immunomodulatory properties. Phage receptor-binding proteins (RBPs), found within their tail apparatus, have been shown to have specific targeting properties through their binding with different bacterial surface receptors, thus enabling the specific suppression of IBD pathobionts (Xu and Wozniak, 2015; Dunne et al., 2021; Federici et al., 2023). Studies targeting adherent-invasive *Escherichia coli* and *Klebsiella pneumoniae* have shown significant pathobiont depletion in DSS-induced models of colitis and attenuation of histopathological and clinical severity of disease following phage therapy intervention (Yin et al., 2024). However, although these results are supported by robust preclinical data, clinical validation of the effectiveness of bacteriophage treatment for IBD is limited, with much of the information available having come from non-IBD settings.

Additionally, phage therapy has been correlated with decreased systemic inflammatory markers such as C-reactive protein (CRP), leukocytes, and erythrocyte sedimentation rates (ESR), as well as decreased inflammatory cytokines and reactive oxygen species (ROS), all of which are key drivers of IBD pathophysiology (Souza et al., 2023). In light of the increased risk of thrombosis in IBD patients, combined therapy regimens utilizing nitric oxide have been highlighted for application owing to its anti-thrombotic properties through decreased platelet adhesion (Gondil et al., 2025). Multi-lytic phage cocktails that target susceptible and resistant *Klebsiella pneumoniae* further validate the suppression of colonization and the mitigation of the severity of colitis, while colonization with clinical IBD-derived strains of bacteria exacerbates inflammation (Wang et al., 2024). Nevertheless, the occurrence of resistance-conferring mutations to each of the phages underlines the importance of using multi-phage formulations to ensure therapeutic efficacy (Federici et al., 2023).

Aside from the use of phages, one other possible approach that can serve as an emerging strategy for treating IBDs is the application of helminth-mediated immunomodulation (Jamtsho et al., 2024). Studies involving *Trichuris suis* ova have shown symptomatic improvement in Crohn’s disease; however, safety concerns have also been reported in the initial studies. Nevertheless, the therapeutic potential has been enhanced by subsequent studies showing the efficacy of *Trichuris muris* ova in reducing TNBS-induced colitis in IL-10−/− murine models after oral administration (Szuba et al., 2024). The use of *Nippostrongylus brasiliensis* (Helminth)-derived extracellular vesicles has shown potent immunomodulatory effects in reducing colitis-associated inflammation and cytokines such as IL-6, IL-1β, IFN-γ, and IL-17A (Li et al., 2025a). *Hymenolepis diminuta* also shows the ability to reduce DNBS-induced colitis by inducing Th2 cell-mediated immunity. The use of *Schistosoma mansoni* ova induces Th2 and inhibits Th1 cell-mediated immunity (Cleenewerk et al., 2020). Similarly, the use of *Ancylostoma caninum* showed the ability to downregulate cytokines such as IL-23, TNF, and IL-1β, and the use of *Schistosoma japonicum* regulated the immune response by reducing pro-inflammatory and increasing anti-inflammatory cytokines (Zhou et al., 2021; Yeshi et al., 2024). Significantly, treatments utilizing helminths have been taken into clinical evaluation, with randomized trials demonstrating variable efficacy, indicating partial but not yet conclusive clinical validation. These findings underscore the potential of helminth-based interventions to modulate immune pathways and offer targeted therapeutic strategies for mitigating colitis-driven inflammation.

## Integrating machine learning and multi-omics approaches in inflammatory bowel disease

Traditional diagnosis methods are often invasive and lack the precision of disease subtype or activity classification. This need for improvement has led to the incorporation of machine learning (ML) and multi-omics data to improve non-invasive diagnosis, mechanism understanding, and personalized therapeutic options. For instance, machine learning (ML) algorithms, by integrating clinical data such as biomarkers from serum, urine, and feces, have proven success rates in distinguishing between CD and UC (Kraszewski et al., 2021). Supervised machine learning approaches in microbiome studies in clinically labeled patients demonstrated significant efficacy (AUC ~0.80) in diagnosing non-invasive IBD, suggesting their robust potential as diagnostic aids in the clinical settings (Manandhar et al., 2021).

Recent studies have demonstrated that ML models of metagenomic and microbiome data can detect minimal but highly predictive microbial signatures. For instance, XGBoost models based on shotgun metagenomics and 16S rRNA analysis have identified a 10-species microbial panel that could distinguish inflammatory bowel disease (IBD) patients from healthy controls effectively, emphasizing the need for cross-platform harmonization and training in AI microbiome studies (Yu et al., 2025). Random Forest models have further validated these models for multi-class and binary classification, identifying important discriminatory species such as *Staphylococcus homini*s, *Porphyromonas endodontalis*, and *Slackia piriformis*, thus implicating microbial inflammatory and metabolic pathways in disease heterogeneity (Mihajlović et al., 2021). Interpretability analysis and correlation studies have further validated that common genera between UC and CD play a role in microbiome-mediated inflammatory pathways (Liñares-Blanco et al., 2022).

Beyond taxonomic and species-level classification, advanced computational approaches have further refined microbial pattern detection. Sequence-based methods using k-mer feature sets and machine learning models have been shown to outperform traditional taxonomic approaches, detecting subtle patterns of microbial association with IBD pathogenesis (Li et al., 2025b). Network analyses of microbial communities, together with machine learning (ML), have also improved the discovery of biomarkers. Pediatric studies have shown decreased microbial alpha diversity, impaired co-occurrence networks, decreased anti-inflammatory species (especially *Faecalibacterium*), and increased pro-inflammatory species. These were associated with clinical markers of inflammation and with predictive ML variables, indicating mechanistic relevance and the possibility of treatment-responsive biomarkers (Luo and Yang, 2025). Whole-metagenome ML models have identified species associated with disease severity, such as *Alistipes shahii* and *Polynucleobacter wianus*, allowing for moderate to high discrimination between Crohn’s disease (CD) and ulcerative colitis (UC) (AUC up to 0.873) and enabling microbiome-based non-invasive diagnostics (Kang et al., 2023).

While these approaches focus primarily on microbial composition, integrating multi-omics data provides deeper functional and mechanistic insights. Multi-omics integration further refines this framework by associating microbial signatures with functional pathways. Analyses using shotgun metagenomics, metatranscriptomics, and metabolomics revealed a Crohn’s disease-specific 20-species microbial signature with high diagnostic performance (AUC = 0.94), together with functional perturbations in microbial fermentation pathways that explained the depletion of butyrate, a key anti-inflammatory SCFA. Functional virulence processes in adherent-invasive *Escherichia coli* (AIEC), including ompA gene expression, were demonstrated to enhance bacterial adherence and macrophage invasion. Mechanistically, *E. coli* aspartate depletion and propionate metabolism were demonstrated to induce virulence gene expression, thus directly connecting microbial metabolism to immune system dysfunction. Notably, these changes were not observed in UC, suggesting the existence of subtype-specific microbiome-mediated pathogenic mechanisms (Serrano-Gómez et al., 2025).

Global multi-omics integration has shown consistently dysregulated species, including *Asaccharobacter celatus*, *Gemmiger formicilis*, and *Erysipelatoclostridium ramosum*, as well as functional perturbations in two-component system pathways and aminoacyl-tRNA synthetases. ML-assisted integration enabled the discovery of globally validated biomarkers with AUROC values ranging from 0.92 to 0.98, thus establishing a connection between microbial dysbiosis and mechanistic host-microbiome pathways (Ning et al., 2023; Tao et al., 2025). Stage-specific integration of microbiome and host transcriptomic data using NetMoss and recursive support vector machine (SVM) classifiers has identified microbial patterns and 161 differentially expressed host genes related to immune and metabolic dysfunction, allowing for precise disease stage stratification and the elucidation of microbiota-host interactions (Tao et al., 2025).

Further mechanistic information has been obtained in the context of IBD complications. In TNBS-induced colitis, the combination of transcriptomic and metabolomic analyses identified dynamic changes in lipid metabolism that were associated with the development of intestinal fibrosis, and six metabolites were identified as biomarkers for early diagnosis (Wu et al., 2023). In UC patients who were cytomegalovirus-positive, the combination of transcriptomic and proteomic analyses using ML led to the identification of eight important biomarkers (such as PPP1R12B, CIRBP, CSNK2A2, and others) and 11 aberrant immune cells, providing information on the mechanisms of virus-induced immune system dysfunction and allowing for the development of predictive models for early diagnosis (Chen et al., 2024).

To further evaluate clinical applicability, large-scale validation and integrative studies have been conducted. Large-scale validation efforts, such as the sbv IMPROVER challenge, validated the utility of machine learning-based metagenomic diagnostics for IBD vs. non-IBD classification but not UC vs. CD, highlighting the importance of functional and multi-omics analyses beyond taxonomic approaches (Khachatryan et al., 2023). Clinical studies that combine the use of microbial, transcriptional, and clinical patient data have also been able to stratify and stage diseases by leveraging machine learning platforms, with promising results in the monitoring and treatment of diseases (Tao et al., 2025).

Despite these advancements, several obstacles currently hinder the use of machine learning in IBD from a clinical standpoint. A primary challenge is the retrospective design of training datasets used for IBD machine learning models, along with an over-reliance on internal validation methods. This can lead to inflated results and overestimation of model capabilities, which are unlikely to perform as well when deployed in clinical practice. Another concern that arises from machine learning applications is the variability between different microbiome sampling, sequencing methods, and data processing protocols, which affects the reproducibility of the results. Furthermore, differences between study cohorts, such as dietary habits, geographic location, type of IBD, and previous treatments received, can also affect the generalizability of IBD machine learning models (Lee et al., 2025; Kubinski et al., 2022).

Overall, these studies demonstrate the potential of machine learning–driven multimodal data integration to enhance non-invasive IBD diagnosis and to elucidate interactions between microbes, metabolic activities, and host immune responses. By simultaneously employing metagenomics, metabolomics, transcriptomics, and different aspects of network medicine combined with cutting-edge aspects of machine learning, highlighting strong potential for precision medicine, although further validation is required for clinical implementation.

## Conclusion and future prospects

Significant progress has been made in the understanding of the complex pathogenesis of IBD, which has fostered the treatment approaches beyond the conventional pharmacological and surgical approaches (Muzammil et al., 2023; Kiilerich et al., 2025). The complex relationship between genetic predisposition, immune system abnormalities, metabolic abnormalities, microbial imbalance, and environmental factors remains an obstacle to the efficacy of monotherapeutic approaches and highlights the need to adopt holistic approaches to treatment. Indeed, the development of microbiome-targeted approaches such as microbial engineering, antimicrobial peptides, bacteriophage-based approaches, diet-based approaches, and genome-editing technologies has helped to improve the ability to restore intestinal homeostasis and identify novel therapeutic approaches (Gubatan et al., 2021; Arnold et al., 2023). More recently, artificial intelligence (AI) is revolutionizing the field of IBD research and clinical management through the integration of complex genomics, transcriptomics, metabolomics, and clinical data to improve the predictability of the disease and the selection of personalized therapeutic approaches (Rajkomar et al., 2019; Miotto et al., 2018). Beyond the realm of diagnostics, AI-based analytical tools have the potential to expedite target discovery, improve therapeutic algorithms, and dynamically monitor disease progression to inform data-driven and precision-based clinical management approaches. Considering the fact that the prevalence of IBD is on the rise worldwide, fueled by the effects of urbanization and environmental changes, the fusion of microbiome science and AI-powered precision medicine is a promising area in the management of IBD in the future. Thus, it is possible to move beyond the management of the disease to the correction of the immunomicrobial imbalance, leading to disease modification.
